# Malnutrition and type 2 diabetes mellitus across diverse global populations: a systematic review of observational evidence

**DOI:** 10.3389/fcdhc.2026.1749421

**Published:** 2026-07-15

**Authors:** Olalekan Chris Akinsulie, Oluwagbemisola Oyin Olukogbe, Babatunde Ibrahim Olowu, Victor Ayodele Aliyu, Ibrahim Idris, Charles Egede Ugwu, Damilola A. Ibirogba, Adesina Abiodun Ridwan, Oluwole Fasakin, Fakorede Bushroh Adeola

**Affiliations:** 1 College of Veterinary Medicine, Washington State University, Pullman, WA, United States; 2 Faculty of Veterinary Medicine, University of Ibadan, Ibadan, Nigeria; 3 Department of Human Nutrition and Dietetics, College of Medicine, University of Ibadan, Ibadan, Nigeria; 4 Faculty of Veterinary Medicine, Usmanu Danfodiyo University, Sokoto, Nigeria; 5 Center for Discoveries in Life Sciences, Coventry University, Conventry, United Kingdom; 6 Department of Product and Engineering, Crintex Solutions LTD, Port Harcourt, Nigeria

**Keywords:** diabetes mellitus, insulin resistance, malnutrition, systematic review, T2DM

## Abstract

**Background:**

Malnutrition, encompassing undernutrition, overnutrition, and micronutrient deficiency represents a global public health challenge with substantial implications for the development and clinical progression of type 2 diabetes mellitus (T2DM). The bidirectional relationship between nutritional status and glycemic regulation remains incompletely characterized across diverse geographic and socioeconomic settings worldwide, warranting a systematic synthesis of the available observational evidence.

**Methods:**

A systematic review was conducted in accordance with the Preferred Reporting Items for Systematic Reviews and Meta-Analyses (PRISMA) guidelines. Searches were conducted across PubMed/MEDLINE, EMBASE, and the Cochrane CENTRAL register from inception through December 2024. Twenty primary studies were included from multiple high-, middle-, and low-income countries across Asia, Europe, Africa, the Americas, and the Middle East, encompassing a total of more than 25,000 participants. Methodological quality was assessed using the Critical Appraisal Skills Programme (CASP) checklist; 45% of studies were assessed as high confidence and 55% as moderate confidence, following structured qualitative domain-level appraisal.

**Results:**

Malnutrition prevalence, assessed by multiple validated instruments, ranged substantially across study populations and settings. Nutritional risk, defined by Geriatric Nutritional Risk Index criteria, was identified in 30.3% of participants in one Chinese cross-sectional study (n=551). Malnutrition prevalence, assessed using heterogeneous validated instruments across clinically and geographically diverse populations, ranged from 18.5% to 83.8% across included studies; this wide range reflects substantial variation in assessment instrument, patient acuity, clinical setting, and population demographics rather than a uniform epidemiological estimate. Indicators of undernutrition — including hypoalbuminemia, low BMI, and micronutrient deficiency — were associated with impaired glycemic regulation, greater complication burden, and elevated mortality risk. Adjusted odds ratios for diabetic retinopathy associated with malnutrition ranged from 1.67 (95% CI: 1.04–2.70) to 2.24 (95% CI: 1.07–4.69) depending on nutritional index used. Severe malnutrition was associated with 8.91-fold higher adjusted mortality odds (95% CI: 1.04–76.18) compared with moderate malnutrition among hospitalized T2DM patients. Conversely, indicators of overnutrition including elevated BMI and increasing waist circumference — were consistently associated with incident T2DM and adverse cardiometabolic profiles across multiple prospective and cross-sectional studies. Early-life famine exposure was associated with elevated adjusted odds of hyperglycemia in adulthood (aOR range: 1.34–1.57), emphasizing the relevance of developmental nutritional programming to adult T2DM risk. A dual burden of malnutrition concurrent undernutrition and overnutrition within the same populations and study samples — was observed across multiple geographic settings, consistent with patterns documented during nutritional transition.

**Conclusion:**

This systematic review identifies broadly consistent observational associations, with variability in association strength and direction by outcome domain and clinical context between malnutrition in both its under- and overnutrition forms and adverse clinical outcomes in T2DM across diverse global populations. These findings should be interpreted as associative rather than causal, given the predominance of observational cross-sectional and case-control designs, substantial heterogeneity in malnutrition definitions and outcome measures, and inherent risks of reverse causality and residual confounding. Future research should prioritize longitudinal and mechanistic study designs to clarify temporal pathways, and contextually appropriate nutritional screening should be integrated into T2DM prevention and management frameworks globally.

**Systematic Review Registration:**

https://www.crd.york.ac.uk/prospero/, identifier, CRD42024559848.

## 1 Background

Malnutrition constitutes a global public health challenge characterized by a dual burden: overnutrition including being overweight and obesity is increasingly prevalent in high- and middle-income countries undergoing nutritional transition, while undernutrition and micronutrient deficiency continue to disproportionately affect populations in low-income settings (1, 2). These two phenotypically distinct forms of malnutrition are not mutually exclusive; resource-constrained environments frequently exhibit both simultaneously, reflecting the epidemiological complexity of contemporary food systems (3). Selected sub-Saharan African settings exemplify this co-occurrence: approximately 216 million children experience stunting or undernutrition in this region yet rising overweight and obesity rates driven by dietary transition are simultaneously documented across urban populations (4). However, this dual burden is by no means confined to sub-Saharan Africa; analogous patterns have been reported across South and Southeast Asia, Latin America, Eastern Europe, and among indigenous and economically transitioning communities globally (5). It is therefore essential to situate the malnutrition–T2DM relationship within a genuinely global framework, drawing on evidence from multiple income settings and geographic regions.

Food insecurity constitutes a proximate structural driver of undernutrition across multiple low- and middle-income settings, arising from land degradation, climate vulnerability, fragile agricultural systems, and resource scarcity (6). In sub-Saharan Africa particularly, these structural determinants interact with rapid urbanization and dietary transition to simultaneously sustain undernutrition while accelerating overnutrition, a dynamic that exemplifies the dual-burden pattern central to this review. Across the life course, the nutritional drivers and consequences of malnutrition vary substantially. In children and adolescents, undernutrition is characterized by stunting, wasting, and inadequate micronutrient intake, which impairs immune function, growth, and metabolic programming, with long-term consequences for cardiometabolic health that persist into adulthood (7). In older adults, disease-related malnutrition arising from reduced dietary intake, malabsorption, and sarcopenia further disrupts protein and energy homeostasis, promotes hormonal dysregulation, and heightens susceptibility to chronic disease (8, 9). These age-dependent nutritional vulnerabilities are not geographically specific; they characterize populations across low-, middle-, and high-income settings, emphasizing the necessity of globally applicable evidence on malnutrition’s relationship with T2DM.

Two mechanistically distinct pathways link malnutrition to susceptibility for type 2 diabetes mellitus (T2DM), and these must be considered separately to avoid conceptual conflation. The undernutrition pathway operates through developmental metabolic programming: early-life nutritional deprivation — whether *in utero*, during infancy, or in childhood — has been associated with impaired pancreatic beta-cell development and reduced functional beta-cell mass, diminished insulin secretory capacity, and dysregulation of the hypothalamic–pituitary–adrenal axis (10). These effects, consistent with the Developmental Origins of Health and Disease (DOHaD) framework, may predispose individuals to T2DM decades after the period of undernutrition has been resolved, a phenomenon referred to as metabolic programming [Barker, 1995]. Evidence from famine studies supports this pathway: Sun et al. (11), included in this review, demonstrated that female participants with fetal-period famine exposure had significantly elevated adjusted odds of hyperglycemia in adulthood (aOR=1.57, 95% CI: 1.25–1.98), with risk increasing monotonically for earlier exposure windows (11). Furthermore, the International Diabetes Federation’s 2025 classification recognizes Type 5 Diabetes — formerly termed malnutrition-related diabetes mellitus (MRDM) as a distinct clinical entity characterized by chronic undernutrition, beta-cell dysfunction in the absence of autoimmunity, and early-onset insulin-requiring diabetes in lean individuals in resource-limited settings. This classification reinforces the biological plausibility of a distinct undernutrition-driven pathway to insulin insufficiency, separate from classical T2DM pathophysiology. By contrast, the overnutrition pathway proceeds through insulin resistance: chronic positive energy balance promotes adipose tissue expansion, particularly visceral adiposity, which in turn drives systemic inflammation, dysregulated adipokine secretion, ectopic lipid deposition in insulin-sensitive tissues, and progressive impairment of insulin signaling (12). This cascade constitutes the principal pathophysiological mechanism linking obesity to T2DM in most included studies from high- and middle-income settings. The methodological separation of these two pathways is critical to interpreting the heterogeneity of findings across this review’s included studies. In children, young adults, older adults, and the elderly, diabetes mellitus has been described as one of the most common sequelae of overnutrition and a related cause of malnutrition (13). Both undernutrition and impaired nutrient absorption or utilization has been observationally associated with increased susceptibility to diabetes mellitus; the type of diabetes mellitus most implicated differs by nutritional exposure pathway and mechanistic context (13). The pathophysiology and pathogenesis of diet-induced diabetes mellitus remain relatively constant, but type-1 diabetes mellitus occurs more frequently in the setting of undernutrition (14), particularly in children and young adults in resource-poor settings (15). In contrast, type 2 diabetes mellitus is more commonly observed in older adults and the elderly, in whom weakened immune function and malabsorption may contribute to nutritional vulnerability and disease progression (12). Against this backdrop, a systematic synthesis of the global evidence on the association between malnutrition and T2DM is warranted. Previous reviews have largely examined specific nutritional exposures or focused on single geographic regions, leaving a gap in comprehensive cross-setting evidence synthesis. The present review aims to address this gap by examining the relationship between malnutrition in both its undernutrition and overnutrition forms and T2DM across diverse populations spanning multiple income settings and geographic regions, with particular attention to glycemic outcomes, clinical complications, quality of life, and cardiovascular dysfunction.

## 2 Methods

This systematic review adhered to the Preferred Reporting Items for Systematic Reviews and Meta-analysis (PRISMA) guidelines.

### 2.1 Review registration

This review was registered on Prospero with ID CRD42024559848.

### 2.2 Research question

This review attempts to answer the following questions: What is the association between malnutrition and type 2 diabetes mellitus, and what clinical outcomes are reported among individuals with coexisting malnutrition and T2DM? What are the long-term health consequences of malnutrition on the development and progression of Type 2 diabetes mellitus?

### 2.3 Search strategy and databases

A comprehensive systematic literature search was conducted across PubMed/MEDLINE, EMBASE, and the Cochrane Central Register of Controlled Trials (CENTRAL) from database inception through December 2024 to identify studies evaluating the association between malnutrition and type 2 diabetes mellitus (T2DM). To maximize methodological rigor, sensitivity, and reproducibility, both controlled vocabulary terms (e.g., Medical Subject Headings [MeSH] for PubMed and Emtree terms for EMBASE) and free-text keywords were employed. Search terms were developed to comprehensively capture all major dimensions of malnutrition, including undernutrition, overnutrition, obesity, overweight, underweight, stunting, wasting, protein-energy malnutrition, micronutrient deficiency, and nutritional imbalance, alongside diabetes-related terms, including type 2 diabetes mellitus, T2DM, insulin resistance, hyperglycemia, glycemic control, and metabolic dysfunction. Boolean operators (“AND,” “OR”), truncation, phrase searching, and database-specific syntax modifications were applied to optimize retrieval across platforms. Searches were limited to human studies published in English. In addition, manual screening of reference lists from eligible studies was conducted to identify potentially relevant studies not captured by electronic searches.

### 2.4 Database-specific search framework

#### 2.4.1 PubMed/MEDLINE

(“Malnutrition”[MeSH] OR malnutrition OR undernutrition OR underweight OR stunting OR wasting OR “protein-energy malnutrition” OR obesity OR overweight OR overnutrition OR “micronutrient deficiency” OR “nutritional deficiency”) AND (“Diabetes Mellitus, Type 2”[MeSH] OR “type 2 diabetes mellitus” OR T2DM OR “insulin resistance” OR hyperglycemia OR “glycemic control”).

#### 2.4.2 EMBASE

(‘malnutrition’/exp OR malnutrition OR undernutrition OR obesity OR overweight OR underweight OR stunting OR wasting OR ‘protein energy malnutrition’ OR ‘nutritional deficiency’ OR ‘micronutrient deficiency’) AND (‘type 2 diabetes mellitus’/exp OR ‘type 2 diabetes mellitus’ OR T2DM OR ‘insulin resistance’ OR hyperglycemia OR ‘glycemic control’).

#### 2.4.3 Cochrane central

(malnutrition OR undernutrition OR obesity OR overweight OR underweight OR stunting OR wasting OR “protein-energy malnutrition” OR “micronutrient deficiency” OR “nutritional deficiency”) AND (“type 2 diabetes mellitus” OR T2DM OR “insulin resistance” OR hyperglycemia OR “glycemic control”).

### 2.5 Inclusion and exclusion criteria

Studies were considered for inclusion if they were primary studies with confirmed cases of T2DM and measured malnutrition rates in human participants of any age and gender. Studies measuring outcomes such as glycemic control (e.g., fasting blood glucose, HbA1c), insulin resistance, or clinical diagnosis of type 2 diabetes mellitus were also included.

Exclusion criteria were set as studies focusing solely on specific medical treatments or interventions for type 2 diabetes mellitus without considering the role of malnutrition. Studies without appropriate control or comparison groups. Studies that do not report relevant outcomes related to the association between malnutrition and type 2 diabetes mellitus. Case reports, editorials, letters, commentaries, and conference abstracts. Animal studies or *in vitro* studies. Finally, non-English studies were excluded unless translations were available.

### 2.6 Quality assessment

Three reviewers independently assessed the methodological quality and risk of bias of each included study using the Critical Appraisal Skills Programme (CASP) checklist appropriate to each study design (16). In accordance with the original intent of the CASP framework, the checklist was applied as a structured qualitative appraisal instrument; no numerical aggregate score was assigned to individual studies. Each study was systematically appraised across ten methodological domains: clarity of research aims and objectives, appropriateness of study design for the research question, rigor and transparency of participant recruitment, validity and reliability of exposure and outcome measurement, adequacy of confounding identification and control, ethical integrity, completeness of outcome reporting, precision and plausibility of results, generalizability of findings to the target population, and overall methodological coherence. Following structured domain-level appraisal, each study was assigned an overall qualitative confidence rating high, moderate, or low reflecting the pattern of assessments across all appraised domains. Studies rated as high confidence demonstrated few or no substantive methodological concerns across all or nearly all domains. Studies rated as moderate confidence exhibited partial limitations in one or more domains, most commonly in confounding adjustment, participant recruitment transparency, or external validity. No included studies were assessed as low confidence. Discrepancies between reviewers were resolved through structured discussion and, where necessary, third-reviewer arbitration. Although no studies were excluded solely based on methodological confidence, appraisal findings were explicitly integrated into the narrative synthesis, with findings from lower-confidence studies interpreted with appropriately greater caution. Study-level domain appraisals are provided in **Supplementary Table 1**.

### 2.7 Data screening and analysis

Two independent reviewers screened the titles and abstracts before assessing the full texts using pre-set eligibility criteria. They then resolved conflicts and reached a consensus through discussion. Where necessary, a third reviewer’s opinion was sought for arbitration.

**Figure 1:**
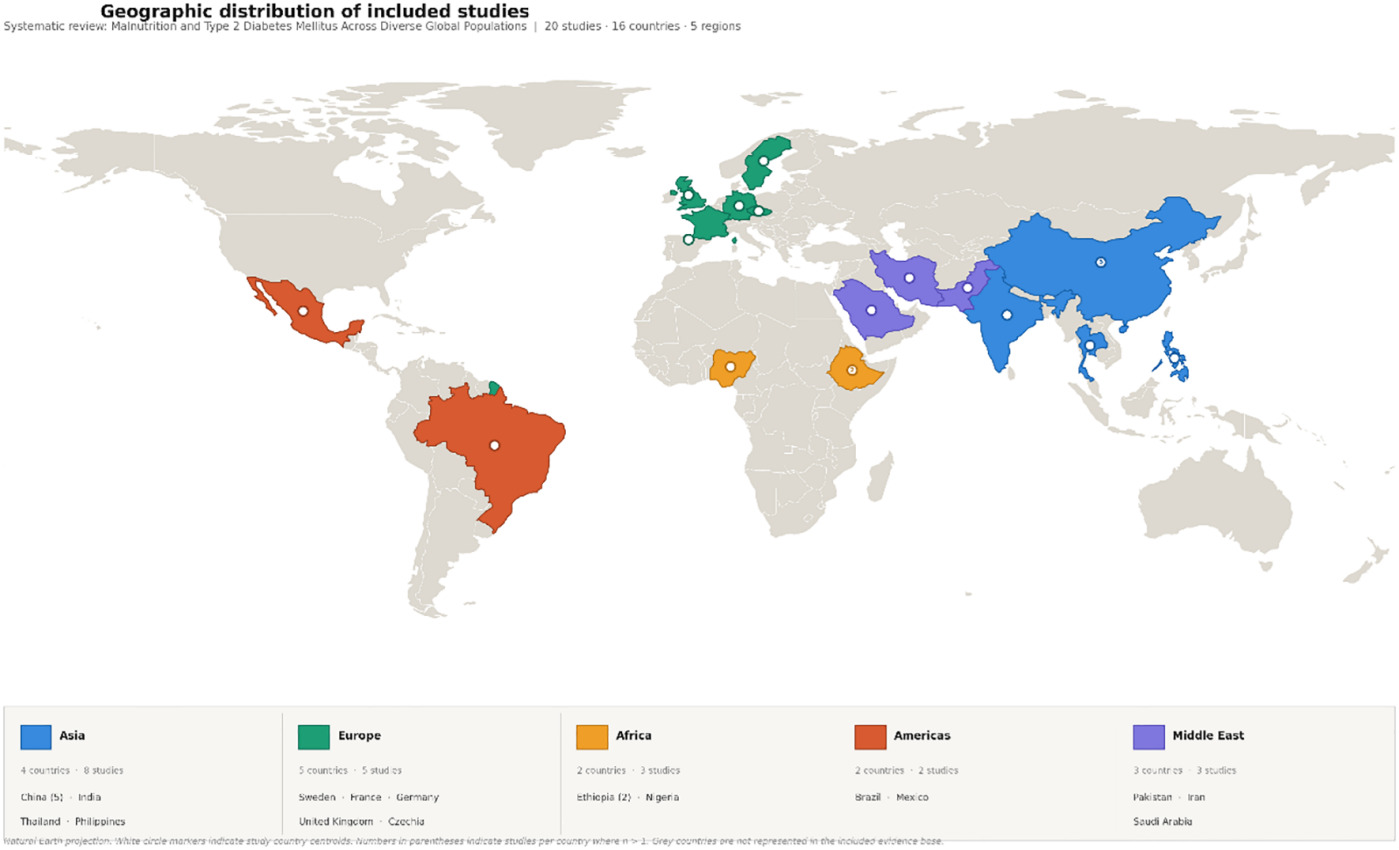
A geographic distribution map of all 20 included studies,.

**Figure 2:**
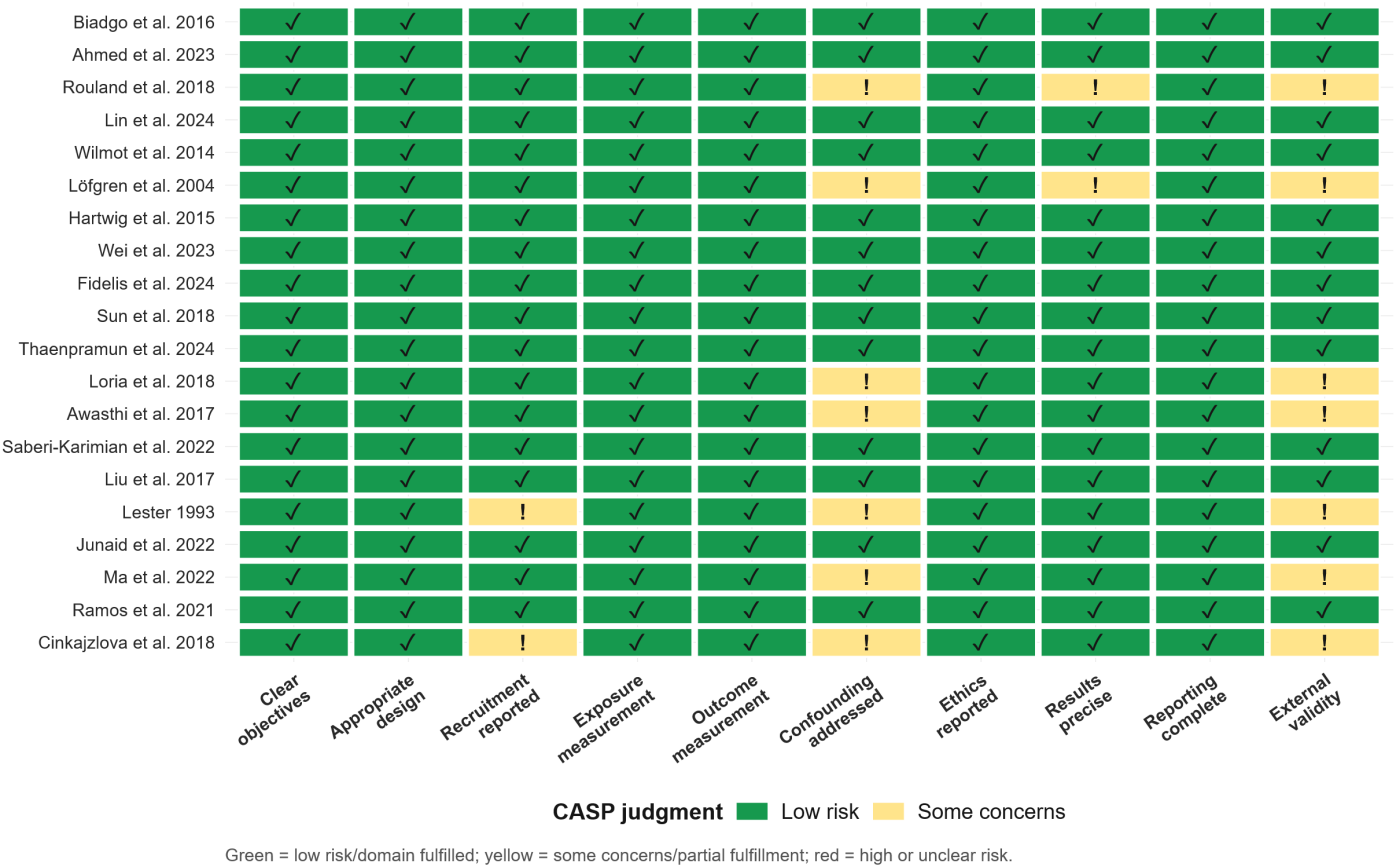
Risk-of-bias summary across included studies assessed using the critical appraisal skills programme (CASP) checklist.

Three reviewers then independently extracted information from the selected studies, including the authors’ names, year, country, study designs, sample size, findings, and end outcomes. First, the study size was stratified by age distribution, as depicted in **Figure 1**. Further, as shown in **Figure 2**, the distribution of the total sample sizes by study design shows a widespread distribution. The violin plot shows the distribution of sample sizes across different study designs, with the wider sections indicating more frequent sample sizes, while narrower sections indicate less frequent ones.

Data abstracted from all studies have been compiled in a matrix table (**Table 1**).

**Table 1:** Outcome summary of malnutrition among T2DM.

Outcome summary of malnutrition among T2DM patients		Studies
Glycemic Control	Insulin resistance	(17, 18, 11, 19, 20)
	Elevated glucose concentration (Poor Glycemic control)	
Poor Nutrition	Increased risk of undernutrition	(8, 19, 21, 22)
	Increased Body Mass Index	(18, 23–28)
Diabetic Complications	Diabetic Nephropathy	(8)
	Diabetic Neuropathy	(29)
	Diabetic Retinopathy	(18, 30)
Reduced Quality of Life	Increased Mortality and Morbidity	(31)
	Slower rates of recovery/Resistance to treatment	(21, 22)
	Low Socio-economic status	(32)
Cardiovascular Dysfunction	Elevated Blood Pressure	(33, 34)

### 2.8 Data synthesis and analytic approach

Prior to data synthesis, a formal prospective assessment of the feasibility of quantitative pooling (meta-analysis) was conducted, in accordance with guidance from the Cochrane Handbook for Systematic Reviews (Version 6.4; Higgins et al., 2023) and the Synthesis Without Meta-analysis (SWiM) reporting guideline (Campbell et al., 2020). Meta-analytic pooling was considered methodologically defensible only if included studies demonstrated sufficient comparability across malnutrition exposure definitions, outcome measures, effect estimate types, and study populations to produce clinically interpretable pooled estimates.

Upon completion of data extraction, the following sources of critical heterogeneity were identified as precluding formal quantitative synthesis. First, malnutrition was operationalized using at least ten distinct validated assessment instruments across the 20 included studies, encompassing the Mini Nutritional Assessment (MNA), Geriatric Nutritional Risk Index (GNRI), Global Leadership Initiative on Malnutrition (GLIM) criteria, Subjective Global Assessment (SGA), Nutritional Risk Index (NRI), Prognostic Nutritional Index (PNI), Controlling Nutritional Status (CONUT) score, early-life famine exposure classification, and anthropometric index composites instruments reflecting related but operationally non-equivalent malnutrition constructs. Second, primary outcomes spanned incident T2DM, glycemic regulation (HbA1c), diabetic retinopathy, all-cause mortality, hospitalization duration, cardiovascular structural parameters, and population-level nutritional transition. Third, no outcome domain was represented by three or more studies sharing compatible exposures, effect estimate types, and populations sufficient to satisfy minimum conditions for interpretable pooling.

**Figure 3:**
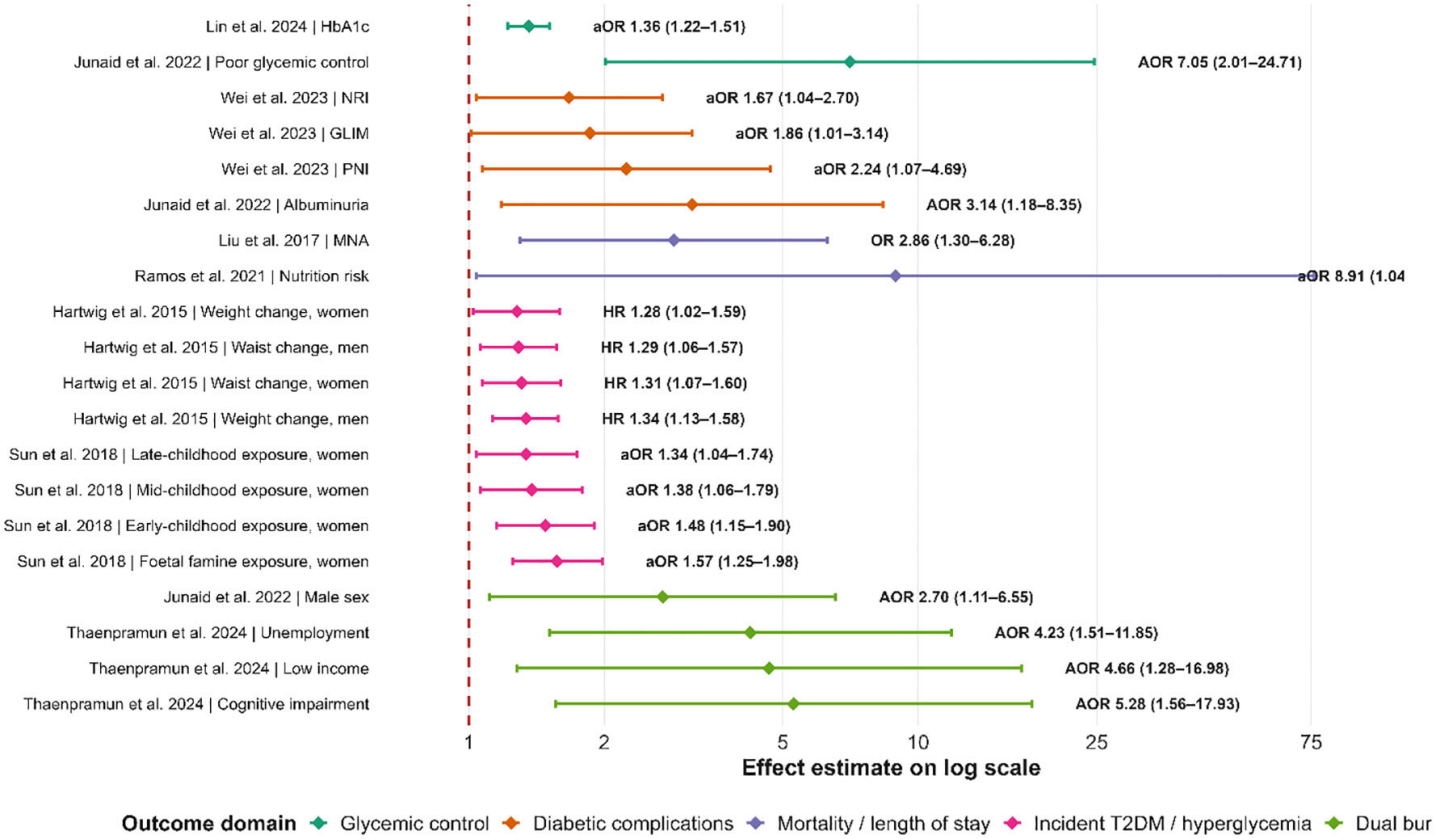
Descriptive display of study-level adjusted effect estimates, and 95% confidence intervals organized by clinical outcome domain. Adjusted odds ratios (aORs) and hazard ratios (HRs) are presented for studies reporting multivariable-adjusted estimates. This figure represents a descriptive visualization of study-level quantitative findings and does not constitute a meta-analytic forest plot; no pooled estimate is implied or presented. Outcome domains displayed: diabetic retinopathy, mortality, glycemic dysregulation, incident T2DM, and malnutrition clinical predictors. *Wei* et al. *(*30*) contributes three estimates from the same study using different nutritional indices (GLIM, NRI, PNI)*.

Under these conditions, meta-analytic pooling would have generated numerically precise but clinically uninterpretable estimates, inconsistent with principles of methodological transparency. Accordingly, a narrative synthesis was conducted following SWiM guidance, with explicit reporting of study-level adjusted effect estimates and 95% confidence intervals where available. Extracted quantitative findings are displayed descriptively by outcome domain in **Figure 3** to facilitate cross-study comparison without implying statistical poolability.

## 3 Results

**Figure 4:**
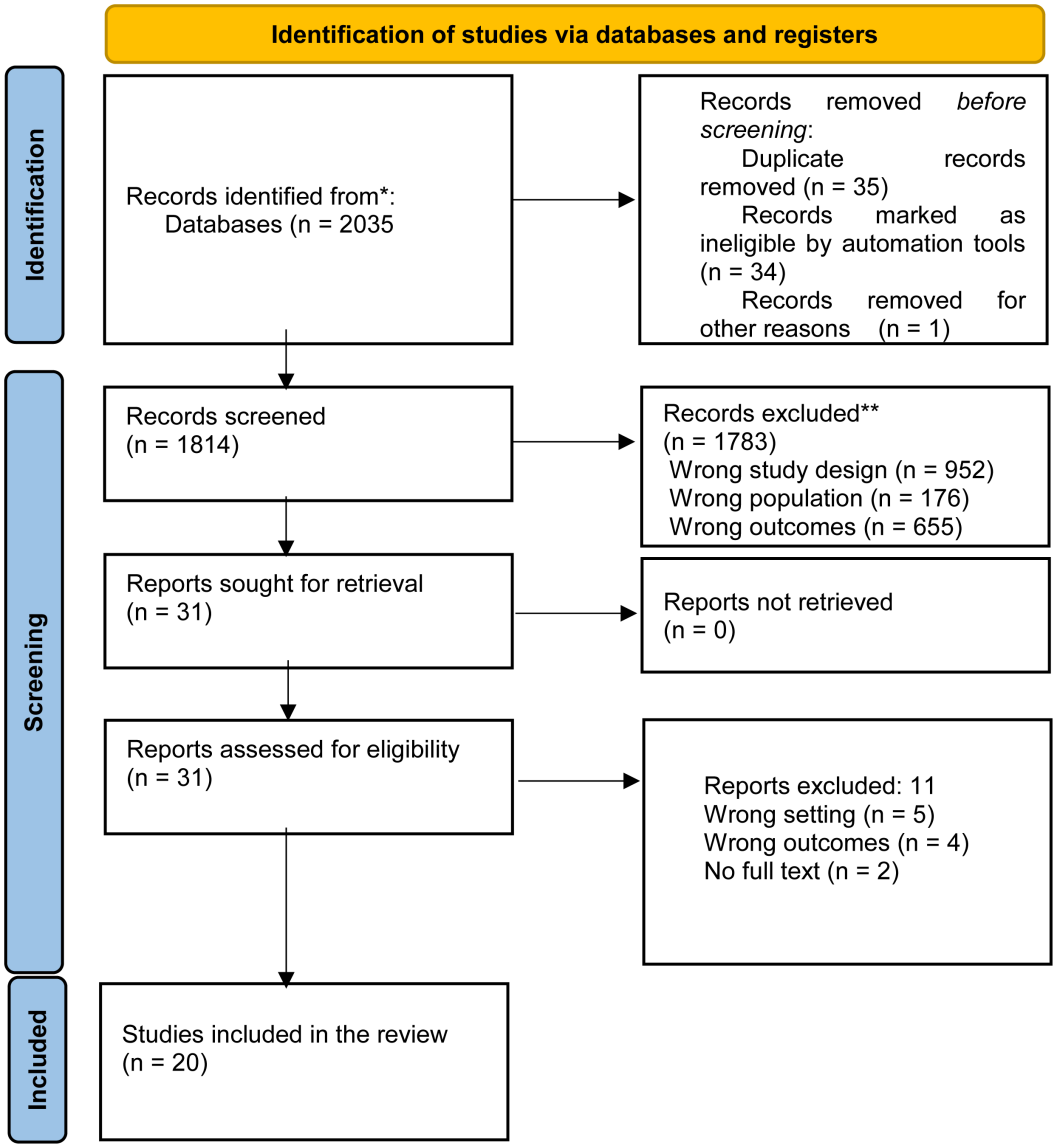
The PRISMA diagram showing the included studies and rationale for selection.

A comprehensive electronic literature search across PubMed/MEDLINE, EMBASE, and Cochrane CENTRAL identified 2,035 records. Prior to abstract-level screening, 221 records were removed: 35 duplicate records, 34 records identified as ineligible by automated screening tools, 151 records excluded following title-only review as clearly outside the scope of the review question, and 1 record removed for other reasons. The remaining 1,814 records were screened at the title and abstract level, of which 1,783 were excluded: 952 for wrong study design, 176 for wrong population, and 655 for wrong outcomes. The 31 remaining reports were retrieved and assessed for full eligibility. Eleven reports were subsequently excluded: 5 for wrong setting, 4 for wrong outcomes, and 2 for unavailability of full text. Twenty studies met all eligibility criteria and were included in the final review (**Figure 4**).

**Figure 5:**
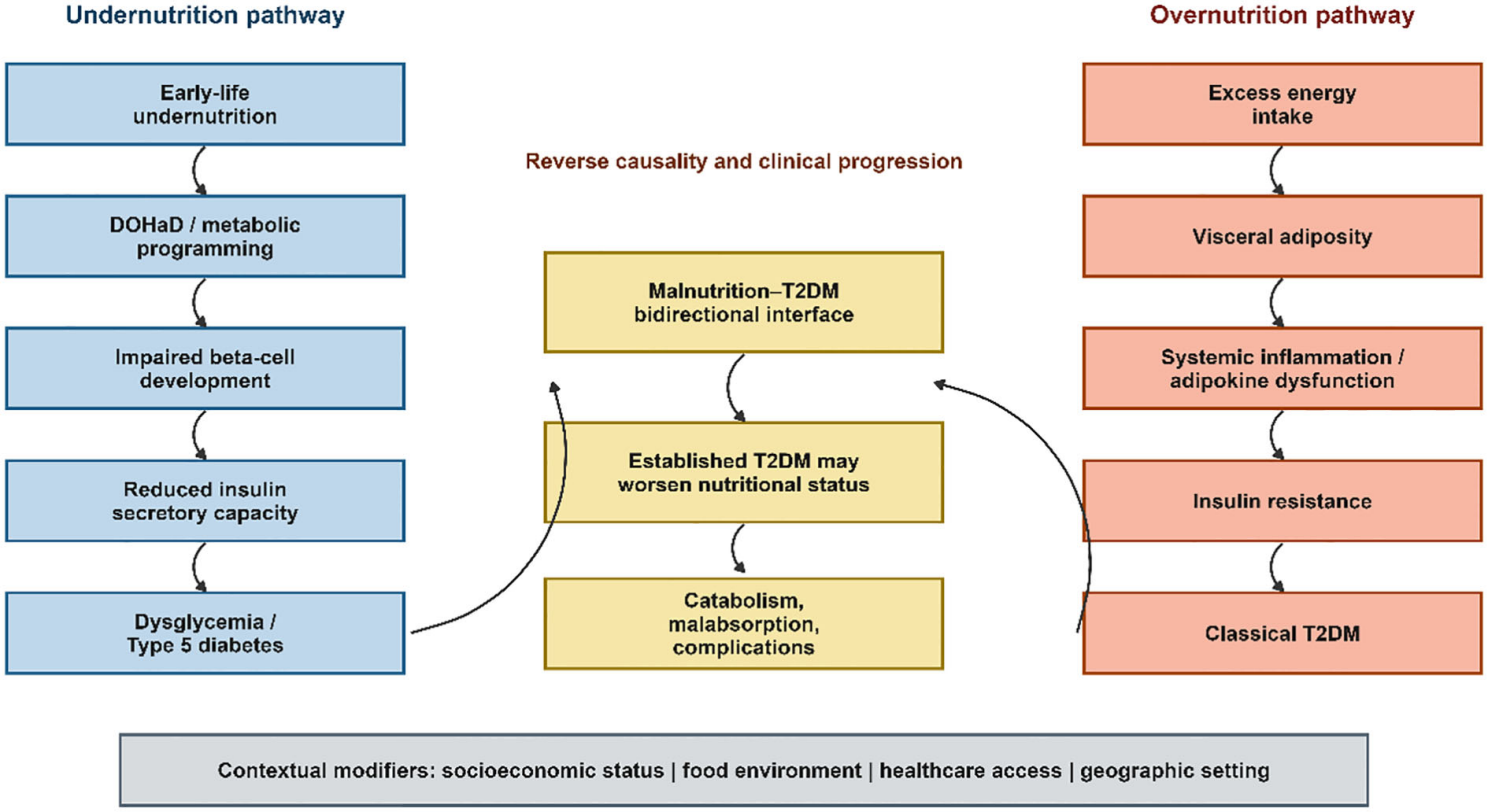
Conceptual framework illustrating the proposed pathways linking undernutrition- and overnutrition-related mechanisms to type 2 diabetes mellitus (T2DM), including beta-cell dysfunction, insulin resistance, and bidirectional effects of T2DM on nutritional status.

Ultimately, only 20 articles met the requirements for inclusion; these were added to the study as shown in **Figure 4**, and the quality assessment process was restarted. The 20 included studies were classified by primary study design as follows: 10 cross-sectional studies (8, 17–20, 25, 30, 32–34), 5 prospective cohort or longitudinal observational studies (11, 23, 27, 29, 31), 4 retrospective observational studies (21, 22, 24, 28), and 1 case-control study (26). The predominance of cross-sectional designs is consistent with patterns documented in nutritional epidemiology and inherently limits causal and temporal inference from pooled findings. Of the included studies, the majority demonstrated moderate-to-high methodological quality, with 9 studies (45%) were assessed as high confidence and 11 studies (55%) as moderate confidence upon structured CASP domain-level appraisal. While most studies exhibited strong methodological rigor, moderate-quality studies commonly showed limitations related to confounding control, participant recruitment strategies, and external generalizability. These methodological differences were considered during interpretation of findings, with lower-quality studies contributing comparatively less certainty to the overall evidence synthesis. The included studies were conducted across 16 countries spanning five geographic regions (Asia, Europe, Africa, the Americas, and the Middle East). **Figure 5** illustrates the geographic distribution of the included studies, while **Table 2** summarizes their characteristics, study design, location, and key findings. The selected articles were published between 1990 and 2023.

**Table 2:** Characteristics, clinical outcomes, and quantitative findings of included studies.

Author (year)	Country	Design/sample	Nutrition measure	Clinical outcome	Quantitative findings/effect size
Biadgo et al., 2016 (17)	Ethiopia	Comparative cross-sectional/Cross-sectional; n=296 (148 T2DM, 148 controls	BMI, WC, WHR; metabolic/hematologic indicators	Glycemic/metabolic profile	T2DM patients had higher BMI (25.4 ± 3.2 vs 23.2 ± 3.1 kg/m2, p<0.001), WC (83.02 ± 12.6 vs 78.67 ± 10.2 cm, p=0.001), WHR (0.88 ± 0.88 vs 0.84 ± 0.66, p<0.001), systolic BP [130 (120-140) vs 110 (110-120), p<0.001], and FBG (163.7 ± 1.33 vs 81.3 ± 1.08 mg/dL, p<0.001).
Ahmed et al., 2023 (8)	Pakistan	Cross-sectional; n=359	Subjective Global Assessment (SGA)	Malnutrition prevalence and associated clinical factors	Mild/moderate malnutrition was 48.2%; severe malnutrition was 10.6%. BMI differed by nutritional status (p<0.01), CAD differed by nutritional status (p=0.015), and BMI correlated negatively with SGA nutritional status (r=-0.351, p<0.01).
Rouland et al., 2018 (29)	France	Prospective observational; n=48; 24-week follow-up	Protein-energy malnutrition using HAS criteria	Diabetic foot ulcer healing	Malnutrition prevalence was 29.2% at baseline and 25.6% at follow-up. Among healed ulcers, 29% were undernourished at inclusion and 17% at follow-up; among non-healed ulcers, 50% and 42%, respectively. Malnutrition was not significantly associated with wound healing or healing duration.
Lin et al., 2024 (18)	China	Cross-sectional; n=551	GNRI-defined malnutrition exposure risk; SHBG and nutrition indicators	Malnutrition exposure risk/glycemic and biochemical indicators	167/551 (30.31%) were classified as having malnutrition exposure risk (GNRI<=98). Independent associations: SHBG OR = 1.04 (95% CI: 1.02-1.05, p<0.001), HbA1c OR = 1.36 (95% CI: 1.22-1.51, p<0.001), hemoglobin OR = 0.96 (95% CI: 0.94-0.97, p<0.001), NAFLD OR = 0.41 (95% CI: 0.23-0.73, p=0.003).
Wilmot et al., 2014 (33)	United Kingdom	Observational pilot; n=40 (20 T2DM, 10 lean controls, 10 obese controls)	Obesity/BMI and cardiometabolic phenotype	Cardiovascular dysfunction	T2DM group BMI was 33.9 ± 5.8 kg/m2. Compared with lean controls, T2DM participants had higher LV mass [85.2 (76.4, 102.9) vs 80.8 (70.9, 85.5) g, p=0.002] and reduced peak early diastolic strain rate [1.51 ± 0.24/s vs 1.97 ± 0.34/s, p=0.001]; strain rate was also reduced vs obese controls [1.78 ± 0.39/s, p=0.042].
Löfgren et al., 2004 (34)	Sweden	Cross-sectional descriptive; n=45	BMI/nutritional vulnerability in elderly patients with diabetes	Hypoglycemia/glycemic control	Mean HbA1c was 5.9 ± 1.1% (range 3.6-8.6%). Fourteen hypoglycemic episodes (blood glucose <=4.0 mmol/L) occurred in 8 patients. BMI was lower in the lower HbA1c tertile than in middle and higher tertiles (p<0.003 and p<0.04).
Hartwig et al., 2015 (23)	Germany	Prospective cohort; n=2,602	Weight and waist circumference change	Incident T2DM	Adjusted HRs: absolute weight change per year - women HR = 1.28 (95% CI: 1.02-1.59), men HR = 1.34 (95% CI: 1.13-1.58); absolute WC change per year - women HR = 1.31 (95% CI: 1.07-1.60), men HR = 1.29 (95% CI: 1.06-1.57).
Wei et al., 2023 (30)	China	Cross-sectional; n=612	GLIM, CONUT, NRI, PNI	Diabetic retinopathy presence and severity	Malnutrition prevalence ranged from 10.0% to 34.3% overall and 16.3% to 45.1% among DR patients. Adjusted ORs for DR: GLIM aOR=1.86 (95% CI: 1.01-3.14, p=0.046), NRI aOR=1.67 (95% CI: 1.04-2.70, p=0.034), PNI aOR=2.24 (95% CI: 1.07-4.69, p=0.032). Adjusted ORs for DR severity: GLIM 1.99, NRI 1.65, PNI 2.51.
Fidelis et al., 2024 (24)	Brazil	Observational repeated survey; n=598 in 2007, n=924 in 2021	BMI and waist circumference	Nutritional transition/excess weight and obesity	Excess weight increased across all seven indigenous peoples. Xikrin do Bacaja excess weight increased from 30.1% to 65.5%. General obesity increased notably in Gaviao (14.4% to 33.1%), Kararao (7.7% to 34.8%), and Xikrin do Bacaja (5.0% to 30.4%).
Sun et al., 2018 (11)	China	Retrospective cohort; n=7,262	Early-life famine exposure	Hyperglycemia/T2DM risk in adulthood	In females, fully adjusted ORs for hyperglycemia were elevated for late-childhood exposure OR = 1.34 (95% CI: 1.04-1.74), mid-childhood OR = 1.38 (95% CI: 1.06-1.79), early-childhood OR = 1.48 (95% CI: 1.15-1.90), and fetal exposure OR = 1.57 (95% CI: 1.25-1.98). In males, early- and late-childhood exposure were associated with lower T2DM risk: OR = 0.65 (95% CI: 0.49-0.86) and OR = 0.74 (95% CI: 0.56-0.98).
Thaenpramun et al., 2024 (32)	Thailand	Cross-sectional; n=287	Mini Nutritional Assessment (MNA)	Poor nutrition/glycemic control	Poor nutritional status prevalence was 47/287 (16.4%; manuscript abstract reports 15%). Adequate glycemic control was not associated with poor nutrition (p=0.67 in abstract; model AOR = 0.47, 95% CI: 0.18-1.23, p=0.12). Risk factors: income <£110 AOR = 4.66 (95% CI: 1.28-16.98), income £110–331 AOR = 7.80 (95% CI: 1.74-34.89), unemployment AOR = 4.23 (95% CI: 1.51-11.85), cognitive impairment AOR = 5.28 (95% CI: 1.56-17.93).
Loria et al., 2018 (25)	Mexico	Observational survey; n=263	BMI and fasting blood glucose	Obesity and diabetes prevalence during socioeconomic transition	In 2000, diabetes prevalence (FBG >125 mg/dL) was 10.6% and obesity prevalence (BMI >=30 kg/m2) was 35.7%. These contrasted with 1962 diabetes prevalence of 2.3% in women and 0% in men under conditions of widespread malnutrition.
Awasthi et al., 2017 (26)	India	Case-control; n=102 (51 cases, 51 controls)	BMI, WC, WHR, WSR	Obesity/T2DM association	BMI >=25 kg/m2 occurred in 55% of cases vs 21.6% controls (p<0.05). High WC occurred in 74.5% cases vs 45.1% controls (p<0.05); high WC odds were 3.56 times higher in diabetics. WSR was significant; WHR was not a sensitive marker, especially in females.
Saberi-Karimian et al., 2022 (27)	Iran	Prospective cohort; n=9,354	Anthropometric indices (WC, BAI, BMI, etc.)	T2DM prediction	T2DM group included 1,461 women and 875 men. In logistic regression, male WC and BAI were associated with T2DM (p<0.001); BAI was the strongest male predictor OR = 1.078 (95% CI: 1.044-1.113). In females, demispan, WC and BAI were significant; WC was strongest OR = 1.085 (95% CI: 1.074-1.095).
Liu et al., 2017 (31)	China	Prospective observational; n=302	MNA	Mortality/hospital stay/diabetic complications	Malnutrition prevalence was 18.5%, risk of malnutrition 33.1%, normal nutrition 48.3%. Independent factors associated with malnutrition included incontinence OR = 3.17 (95% CI: 1.08-9.36), diabetic microvascular complications OR = 2.22 (95% CI: 1.06-4.61), and ADL dependence OR = 11.6 (95% CI: 5.10-26.5). Malnutrition independently predicted mortality OR = 2.86 (95% CI: 1.30-6.28, p=0.009), and LOS was longer in malnourished patients (p=0.003).
Lester, 1993 (28)	Ethiopia	Retrospective observational; n=1,386 adults in manuscript table; source clinic records included 1,835 diabetic patients	BMI/undernutrition; MRDM criteria	Malnutrition-related diabetes mellitus (MRDM)	Among patients aged 15–30 years at onset, 41.3% had BMI<19 kg/m2 at diagnosis; 55% of these had normal weight before symptoms. Among 1,604 patients >18 years at diagnosis, only 21/1,116 with known previous weight had BMI<19 kg/m2 before symptoms. The study found no convincing cases of MRDM fulfilling published criteria.
Junaid et al., 2022 (19)	Nigeria	Cross-sectional; n=192 (96 T2DM, 96 controls)	MNA-SF, serum albumin, BMI	Malnutrition in elderly T2DM	Among elderly with T2DM vs controls: hypoalbuminemia 79.2% vs 25.0% (p<=0.001); overweight/obesity 58.3% vs 24.0%; underweight 16.7% vs 4.2%; MNA-SF malnutrition 7.3% vs 0%, at risk 42.7% vs 16.7% (p<=0.001). Predictors: male gender AOR = 2.70 (95% CI: 1.11-6.55, p=0.028), albuminuria AOR = 3.14 (95% CI: 1.18-8.35, p=0.022), poor glycemic control AOR = 7.05 (95% CI: 2.01-24.71, p=0.002).
Ma et al., 2022 (21)	China	Retrospective observational; n=307 PTB-T2DM	MNA, NRS, BMI, albumin, prealbumin, transferrin, anthropometry	Malnutrition risk in PTB-T2DM	Malnutrition incidence was 40.07% (123/307). Multivariable analysis identified drinking, divorced status, weight<45 kg, BMI<18.5 kg/m2, NRS>=3, Hb<106 g/L, albumin<29 g/L, PA<48 umol/L, transferrin<1.37 mmol/L, FEV1>=67.90%, and RV<2.89% as independent risk factors (all p<0.05).
Ramos et al., 2021 (22)	Philippines	Retrospective observational; n=439	Nutrition risk level/malnutrition degree	Length of stay and mortality	Malnutrition prevalence was 83.8%: 50.3% moderately malnourished and 33.5% severely malnourished. Severe malnutrition was associated with LOS longer by 2.2 days vs well-nourished after controlling for BMI (95% CI: 0.49-3.95, p=0.012). Deaths occurred in 6.1% of severely malnourished and 0.5% of moderately malnourished patients; severe malnutrition had higher mortality odds vs moderate malnutrition aOR=8.91 (95% CI: 1.04-76.18, p=0.046).
Cinkajzlova et al., 2018 (20)	Czech Republic	Cross-sectional comparative; T2DM-obesity group n=40; multiple comparator groups T2DM-obesity group n=40; multiple comparator groups	Obesity, T2DM, anorexia, fasting, realimentation; ANGPTL3/4	Metabolic regulation disruption	ANGPTL4 was higher in obese subjects without/with T2DM than controls: 94.50 ± 9.51 and 134.19 ± 7.69 vs 50.34 ± 4.22 ng/mL (p<0.001). ANGPTL4 was lower in anorexia nervosa vs controls: 38.22 ± 4.48 vs 65.80 ± 7.98 ng/mL (p=0.002). BMI independently predicted ANGPTL3, while BMI and HbA1c independently predicted ANGPTL4 (adjusted R = 0.552, p<0.001).

ADL, activities of daily living; ANGPTL, angiopoietin-like protein; aOR/AOR, adjusted odds ratio; BAI, Body Adiposity Index; BMI, body mass index; BP, blood pressure; CAD, coronary artery disease; CI, confidence interval; CONUT, Controlling Nutritional Status; DR, diabetic retinopathy; FBG/FBS, fasting blood glucose/sugar; FEV1, forced expiratory volume in 1 second; GLIM, Global Leadership Initiative on Malnutrition; GNRI, Geriatric Nutritional Risk Index; Hb, hemoglobin; HbA1c/GHB, glycated hemoglobin; HR, hazard ratio; LOS, length of stay; MNA, Mini Nutritional Assessment; MNA-SF, Mini Nutritional Assessment-Short Form; MRDM, malnutrition-related diabetes mellitus; NAFLD, non-alcoholic fatty liver disease; NRI, Nutritional Risk Index; NRS, Nutritional Risk Screening; OR, odds ratio; PA, prealbumin; PNI, Prognostic Nutritional Index; PTB-T2DM, pulmonary tuberculosis with type 2 diabetes mellitus; RV, residual volume; SGA, Subjective Global Assessment; SHBG, sex hormone-binding globulin; T2DM, type 2 diabetes mellitus; WC, waist circumference; WHR, waist-to-hip ratio; WSR, waist-to-stature ratio.

### 3.1 Outcome summary of malnutrition among T2DM patients

Study-level quantitative findings, outcome domains, and effect measures for all 20 included studies are summarized in **Table 1**, organized by clinical outcome category. Three studies reported significant associations with elevated glucose concentration, and two studies showed that malnutrition is associated with insulin resistance (11, 17–20). Eleven studies discussed poor nutrition outcomes, which were increased risk of undernutrition, protein-energy malnutrition, and increased body mass index. Four studies reported a statistically significant association between undernutrition and T2DM, such as underweight and stunting (8, 19, 21, 22). Seven studies demonstrated a significant correlation between T2DM and increasing body mass index (18, 23–28). Four studies reported associations between malnutrition and diabetic microvascular complications, encompassing diabetic nephropathy, peripheral neuropathy, and retinopathy (8, 18, 29, 30). These associations are observational in nature; causal directionality cannot be established from the available cross-sectional and retrospective study designs. For reduced quality of life problems, four articles showed that malnutrition is associated with T2DM (21, 22, 31, 32). Furthermore, two studies showed that T2DM patients with malnutrition had elevated blood pressure (33, 34).

## 4 Discussion

This systematic review synthesized observational evidence from 20 primary studies conducted across Asia (China, India, Thailand), Europe (Sweden, France, Germany, United Kingdom, Czech Republic), Africa (Ethiopia, Nigeria), the Americas (Brazil, Mexico) AND Asia (China, India, Thailand, Philippines) and the Middle East (Pakistan, Iran, Saudi Arabia). The geographic distribution of included studies reflects a global, rather than regionally focused, evidence base, and findings should be interpreted as reflecting patterns across diverse income and epidemiological contexts. Malnutrition operationalized across studies using a range of validated instruments including the Mini Nutritional Assessment (MNA), Geriatric Nutritional Risk Index (GNRI), Subjective Global Assessment (SGA), and Global Leadership Initiative on Malnutrition (GLIM) criteria was consistently associated with adverse clinical outcomes in individuals with T2DM. However, the predominance of cross-sectional and case-control designs across included studies limits causal inference and temporal attribution, and all associations reported herein should be interpreted accordingly.

### 4.1 Glycemic control

Across included studies, malnutrition was associated with impaired glycemic regulation through multiple mechanisms, with both undernutrition and overnutrition demonstrating distinct relationships with glycemic indices. Lin et al. (2024, n=551, China) found that malnutrition exposure risk defined by a GNRI ≤1–98 and present in 30.3% of participants was independently associated with elevated HbA1c levels (adjusted OR = 1.36, 95% CI: 1.22–1.51, p<0.001), suggesting that nutritional insufficiency correlates with worse chronic glycemic regulation (18). Separately, Rajamanickam et al. (35) observed that individuals with low BMI demonstrated higher HbA1c, random blood glucose, and insulin concentrations relative to those with normal BMI, alongside altered hematological and cytokine profiles, indicating that undernutrition-associated glycemic dysregulation may operate through immunometabolic pathways (35). Among elderly T2DM patients in Thailand (n=287), poor nutritional status was prevalent in 16.4% of participants but was not independently associated with adequacy of glycemic control after adjustment (AOR = 0.47, 95% CI: 0.18–1.23, p=0.12), suggesting that the nutritional–glycemic association may be modified by age and clinical context (32). Furthermore, Löfgren et al. (2004, n=45, Sweden) documented a mean HbA1c of 5.9 ± 1.1%, with hypoglycemic episodes occurring in 8 patients, and lower BMI associated with lower HbA1c tertile (p<0.003), showing the vulnerability of nutritionally compromised patients to iatrogenic glycemic instability. These findings collectively suggest that nutritional status particularly undernutrition, is associated with glycemic dysregulation in T2DM, although directionality and magnitude vary substantially by setting, age group, and malnutrition definition.

### 4.2 Poor nutrition and the dual burden

The included studies collectively demonstrate a dual-burden pattern of malnutrition, the co-occurrence of undernutrition and overnutrition within the same populations or clinical samples — consistent with nutritional transition dynamics documented globally. Junaid et al. (2022, n=192, Nigeria) found that among elderly T2DM patients, 58.3% were overweight or obese while 16.7% were simultaneously underweight, and hypoalbuminemia was present in 79.2% compared with 25.0% in controls (p ≤ 0.001); predictors of malnutrition included poor glycemic control (AOR = 7.05, 95% CI: 2.01–24.71, p=0.002) and male gender (AOR = 2.70, 95% CI: 1.11–6.55, p=0.028) (19). This pattern mirrors the nutritional transition documented among indigenous Maya populations in Mexico, where diabetes prevalence rose from 2.3% in women and 0% in men under conditions of widespread undernutrition in 1962 to 10.6% under conditions of rising obesity by 2000 (25). Similarly, in Amazonian indigenous communities in Brazil, excess weight increased across all seven peoples studied between 2007 and 2021, with general obesity rising from 5.0% to 30.4% in some groups (24). In the context of overnutrition, Hartwig et al. (2015, n=2,602, Germany) found that annual weight gain was associated with incident T2DM in both women (HR = 1.28, 95% CI: 1.02–1.59) and men (HR = 1.34, 95% CI: 1.13–1.58), and that annual waist circumference increase was similarly associated across both sexes (women: HR = 1.31, 95% CI: 1.07–1.60; men: HR = 1.29, 95% CI: 1.06–1.57), underscoring the longitudinal relationship between adiposity progression and T2DM incidence (23). These converging trajectories across multiple global settings reinforce the epidemiological relevance of the dual-burden paradigm beyond any single region.

### 4.3 Diabetic complications

Malnutrition was associated with greater prevalence and severity of multiple T2DM-related microvascular complications across included studies, though directionality cannot be established from available cross-sectional and retrospective data. Wei et al. (2023, n=612, China) found that malnutrition prevalence depending on nutritional index applied ranged from 10.0% to 34.3% overall and from 16.3% to 45.1% among patients with diabetic retinopathy (DR). After multivariable adjustment, the GLIM criteria identified malnutrition with an adjusted OR for DR of 1.86 (95% CI: 1.01–3.14, p=0.046); the Nutritional Risk Index (NRI) yielded an aOR of 1.67 (95% CI: 1.04–2.70, p=0.034); and the Prognostic Nutritional Index (PNI) yielded an aOR of 2.24 (95% CI: 1.07–4.69, p=0.032) (30). Among hospitalized Filipino T2DM patients (n=439), Ramos et al. (22) found that malnutrition prevalence was 83.8%, and that severe malnutrition was independently associated with higher mortality odds compared with moderate malnutrition (aOR=8.91, 95% CI: 1.04–76.18, p=0.046; noting that the extremely wide confidence interval reflects small event counts in this retrospective sample [deaths in 6.1% of severely vs 0.5% of moderately malnourished patients] and should be interpreted with caution) and with a length-of-stay extension of 2.2 days (95% CI: 0.49–3.95, p=0.012) (22). Liu et al. (2017, n=302, China) found that malnutrition present in 18.5% of elderly T2DM patients independently predicted mortality (OR = 2.86, 95% CI: 1.30–6.28, p=0.009) and was associated with longer hospital stays (p=0.003) after adjustment for diabetic microvascular complications (OR = 2.22, 95% CI: 1.06–4.61) and functional dependency (OR = 11.6, 95% CI: 5.10–26.5) (31). Importantly, Rouland et al. (2018, n=48, France) found no statistically significant association between malnutrition and diabetic foot ulcer healing outcomes, suggesting that malnutrition’s effect on complication severity may not be uniform across all complication types (29). This variability emphasizes the importance of complication-specific analyses in future research.

### 4.4 Reduced quality of life

Across included studies, malnutrition was consistently associated with indicators of reduced quality of life among individuals with T2DM, including greater morbidity burden, prolonged hospitalization, and impaired physical and mental health status. These associations, derived from observational and retrospective study designs, do not establish a causal or mechanistic direction; the possibility that severe T2DM independently contributes to nutritional deterioration cannot be excluded from the available evidence. For people with T2DM, the primary objective of early diagnosis and therapy is to improve quality of life (36). The findings reported that malnutrition is associated with prolonged hospital stays and higher mortality, significantly impacting patients’ quality of life​​ (22, 31). Further, socio-economic factors such as low income, unemployment, and cognitive impairment were identified as predictors of poor nutrition and reduced quality of life in elderly diabetic patients​ (32). These reports align with our findings that malnourished T2DM patients face challenges in maintaining a high quality of life.

### 4.5 Cardiovascular dysfunction

Preliminary observational evidence suggests an association between adverse cardiometabolic profiles and malnutrition in individuals with T2DM, though available data remain limited. Wilmot et al. (2014; n=40, United Kingdom) reported that young adults with T2DM exhibited higher left ventricular mass and reduced peak early diastolic strain rate compared with lean controls — structural and functional findings consistent with early cardiometabolic deterioration. These findings, derived from a small pilot study (n=40), should be interpreted with caution pending replication in larger, adequately powered samples Additionally, malnutrition is associated with non-alcoholic fatty liver disease (NAFLD), and this is associated with elevated cardiovascular risk (37). Other risk factors include hypoalbuminemia, low BMI, poor nutritional markers (21), and changes in weight and obesity markers (23). These findings show how malnutrition influenced cardiovascular dysfunction.

## 5 Limitation

This systematic review is subject to several methodological limitations that must be considered during interpretation of findings.

First, the predominance of cross-sectional and retrospective observational study designs across the included literature limits causal and temporal inference. Most included studies assessed nutritional status in individuals with established T2DM, precluding determination of whether malnutrition antedates disease development or represents a downstream consequence of metabolic and clinical deterioration. The possibility of bidirectional causation whereby T2DM worsens nutritional status through glycosuric caloric loss, anorexia, and metabolic dysregulation, while malnutrition concurrently impairs glycemic regulation cannot be excluded from the available observational evidence.

Second, substantial heterogeneity in malnutrition definitions and assessment instruments across included studies prevented formal meta-analytic pooling and limits direct cross-study comparability. The reported malnutrition prevalence range (18.5%–83.8%) reflects variation in instrument, clinical setting, patient acuity, and population demographics rather than a coherent epidemiological parameter. As described in the Methods, narrative synthesis was adopted on prospective methodological grounds.

Third, residual confounding is a pervasive concern. Even studies reporting multivariable-adjusted estimates could not fully account for socioeconomic determinants, comorbidity burden, medication use, dietary behavior, and physical activity — all of which may independently confound the malnutrition–T2DM association.

Fourth, the geographic distribution of included studies — concentrated in East Asia (five Chinese studies, 25%) — limits generalizability to populations from South and Central Asia, the Pacific Islands, indigenous North American communities, and high-income North American settings. English-language restrictions may additionally have introduced reporting bias.

Fifth, two included studies [Rouland et al. (29); Thaenpramun et al. (32)] reported no statistically significant associations between malnutrition and their primary outcomes after multivariable adjustment. Furthermore, the potential for publication bias — whereby studies reporting null findings may be less likely to have been indexed — cannot be excluded and may contribute to apparent consistency of positive associations in the included literature.

Despite these limitations, the convergence of broadly consistent associative evidence across geographically, clinically, and methodologically diverse studies strengthens confidence in the overall directionality of the malnutrition–T2DM relationship, even in the absence of pooled estimates or causal attribution.

## 6 Conclusion

This systematic review synthesizes observational evidence from 20 primary studies conducted across 16 countries, encompassing diverse geographic, clinical, and socioeconomic contexts. The available evidence indicates that malnutrition across its undernutrition and overnutrition forms is broadly associated with adverse clinical outcomes, although the direction and magnitude of associations vary by outcome domain, malnutrition definition, and clinical context, and not all examined associations reached statistical significance among individuals with type 2 diabetes mellitus (T2DM), including impaired glycemic regulation, elevated complication burden, reduced quality of life, and cardiovascular dysfunction. Effect sizes from included quantitative studies are clinically meaningful: malnutrition was associated with adjusted odds of diabetic retinopathy ranging from 1.67 to 2.24 (depending on nutritional index applied), adjusted odds of mortality 8.91 times higher in severely versus moderately malnourished hospitalized patients, and annual weight gain associated with incident T2DM hazard ratios of 1.28–1.34 across sexes. Nevertheless, these findings must be interpreted with caution and appropriately qualified. The predominance of cross-sectional and retrospective observational study designs across the included literature inherently limits temporal inference: it is not possible to establish from available evidence whether malnutrition precedes T2DM development, whether established T2DM worsens nutritional status through metabolic and clinical mechanisms, or whether both relationships operate concurrently. Reverse causality and residual confounding remain important methodological constraints that cannot be fully excluded given the evidence base synthesized here.

Substantial heterogeneity in malnutrition definitions, assessment instruments, outcome measures, and study populations further restricts direct cross-study comparability and limits the generalizability of specific findings. As described in the Methods, formal quantitative pooling was not undertaken given the extensive clinical and methodological heterogeneity across included studies in malnutrition exposure definitions, outcome measures, and effect estimate types; the narrative synthesis presented herein reflects the breadth and directionality of available observational evidence rather than a pooled or generalizable effect estimate. Additionally, the geographic distribution of included studies concentrated in East Asia (25%) and sub-Saharan Africa (15%), with limited representation from Central Asia, West Africa, the Pacific Islands, and high-income North American settings introduces selection and reporting biases that may limit global representativeness. Future research should prioritize well-designed prospective cohort studies and longitudinal investigations that permit temporal sequencing of nutritional exposures and T2DM outcomes, mechanistic research to elucidate the biological pathways linking undernutrition-related beta-cell dysfunction and overnutrition-related insulin resistance to glycemic dysregulation, and population-specific intervention trials testing the impact of targeted nutritional programs on T2DM prevention and clinical outcomes. From a public health perspective, the integration of validated, routine nutritional assessment into T2DM prevention and management pathways represents a scalable, low-cost strategy with the potential to reduce clinical burden across diverse settings. In low-resource contexts characterized by persistent undernutrition, priority interventions should address food security and micronutrient supplementation.

In settings undergoing nutritional transition with rising overweight and obesity, preventive frameworks should emphasize reduction of energy-dense dietary patterns, promotion of physical activity, and strengthening of metabolic risk screening. These strategies are not mutually exclusive and must be implemented in contextually responsive, population-specific ways.

## Data Availability

The datasets presented in this study can be found in online repositories. The names of the repository/repositories and accession number(s) can be found in the article/**Supplementary Material**.
