# Supplementary material for: Malnutrition and type 2 diabetes mellitus across diverse global populations: a systematic review of observational evidence
Source: Front Clin Diabetes Healthc. 2026 Jul 15;7:1749421. doi: 10.3389/fcdhc.2026.1749421 (PMC13439865; doi:10.3389/fcdhc.2026.1749421)
Supplement: Supplementary file 1 [file Table1.docx]

**Supplementary Table S1**

**Structured Qualitative Domain-Level Methodological Appraisal of Included Studies**

**Methods:** Quality appraisal was conducted independently by three reviewers using the Critical Appraisal Skills Programme (CASP) checklist, applied as a structured qualitative appraisal framework in accordance with CASP Programme guidance. No numerical score was assigned to individual studies. Each study was appraised across ten methodological domains (Q1–Q10), with each domain rated **High (H)**, **Moderate (M)**, or **Low (L)** confidence based on reviewer judgment and consensus. An overall confidence rating was assigned reflecting the pattern of domain-level assessments. Discrepancies were resolved by structured discussion or third-reviewer arbitration.

**Key: H** = High confidence (criterion well met); M = Moderate confidence (partial limitation); L = Low confidence (substantial limitation).

| **Study (Author, Year)** | **Country** | **Primary Design** | **Q1** | **Q2** | **Q3** | **Q4** | **Q5** | **Q6** | **Q7** | **Q8** | **Q9** | **Q10** | **Overall Confidence** |
| --- | --- | --- | --- | --- | --- | --- | --- | --- | --- | --- | --- | --- | --- |
| Biadgo et al., 2016 | Ethiopia | Cross-sectional | **H** | **H** | **H** | **H** | **H** | **M** | **H** | **H** | **H** | **M** | **Moderate** |
| Ahmed et al., 2023 | Pakistan | Cross-sectional | **H** | **H** | **H** | **H** | **H** | **H** | **H** | **H** | **H** | **M** | **High** |
| Rouland et al., 2018 | France | Prospective observational | **H** | **H** | **M** | **H** | **H** | **M** | **H** | **H** | **M** | **H** | **Moderate** |
| Lin et al., 2024 | China | Cross-sectional | **H** | **H** | **H** | **H** | **H** | **H** | **H** | **H** | **H** | **M** | **High** |
| Wilmot et al., 2014 | UK | Cross-sectional (pilot) | **H** | **M** | **M** | **H** | **H** | **M** | **H** | **H** | **M** | **M** | **Moderate** |
| Löfgren et al., 2004 | Sweden | Cross-sectional | **H** | **H** | **M** | **H** | **H** | **L** | **H** | **H** | **M** | **M** | **Moderate** |
| Hartwig et al., 2015 | Germany | Prospective cohort | **H** | **H** | **H** | **H** | **H** | **H** | **H** | **H** | **H** | **H** | **High** |
| Wei et al., 2023 | China | Cross-sectional | **H** | **H** | **H** | **H** | **H** | **H** | **H** | **H** | **H** | **M** | **High** |
| Fidelis et al., 2024 | Brazil | Repeated survey | **H** | **H** | **H** | **M** | **H** | **M** | **H** | **H** | **H** | **M** | **Moderate** |
| Sun et al., 2018 | China | Retrospective cohort | **H** | **H** | **H** | **H** | **H** | **H** | **H** | **H** | **H** | **H** | **High** |
| Thaenpramun et al., 2024 | Thailand | Cross-sectional | **H** | **H** | **H** | **H** | **H** | **H** | **H** | **H** | **H** | **M** | **High** |
| Loria et al., 2018 | Mexico | Repeated survey | **H** | **H** | **M** | **M** | **H** | **L** | **H** | **H** | **H** | **M** | **Moderate** |
| Awasthi et al., 2017 | India | Case-control | **H** | **H** | **H** | **H** | **H** | **H** | **H** | **H** | **H** | **M** | **High** |
| Saberi-Karimian et al., 2022 | Iran | Prospective cohort | **H** | **H** | **H** | **H** | **H** | **H** | **H** | **H** | **H** | **H** | **High** |
| Liu et al., 2017 | China | Prospective observational | **H** | **H** | **M** | **H** | **H** | **M** | **M** | **H** | **M** | **H** | **Moderate** |
| Lester, 1993 | Ethiopia | Retrospective | **H** | **H** | **H** | **M** | **H** | **L** | **L** | **H** | **M** | **H** | **Moderate** |
| Junaid et al., 2022 | Nigeria | Cross-sectional | **H** | **H** | **H** | **H** | **H** | **H** | **H** | **H** | **H** | **H** | **High** |
| Ma et al., 2022 | China | Retrospective | **H** | **H** | **M** | **H** | **H** | **M** | **H** | **H** | **M** | **H** | **Moderate** |
| Ramos et al., 2021 | Philippines | Retrospective | **H** | **H** | **H** | **M** | **H** | **H** | **H** | **H** | **M** | **M** | **High** |
| Cinkajzlová et al., 2018 | Czech Republic | Cross-sectional | **H** | **H** | **M** | **H** | **H** | **M** | **H** | **H** | **M** | **M** | **Moderate** |

**CASP Criteria Reference**

| **Domain** | **Label** | **Appraisal Question** |
| --- | --- | --- |
| **Q1** | Aims | Was there a clear and focused research question or aim? |
| **Q2** | Design | Was the study design appropriate for the research question? |
| **Q3** | Recruitment | Was the method of participant recruitment/selection acceptable? |
| **Q4** | Exposure | Were exposures/interventions accurately measured to minimise bias? |
| **Q5** | Outcome | Were outcomes accurately measured to minimise bias? |
| **Q6** | Confounding | Were major confounding factors identified and adequately controlled? |
| **Q7** | Ethics | Was ethical approval and participant consent addressed? |
| **Q8** | Reporting | Were the results clearly and completely reported? |
| **Q9** | Precision | Was the precision of results reported (e.g., confidence intervals, p-values)? |
| **Q10** | Generalisability | Are the results applicable and generalisable to the study population? |

Reference: CASP (2025). Critical Appraisal Skills Programme. <https://casp-uk.net/>

**Summary of Methodological Confidence Ratings**

Of the 20 included studies, 9 studies (45%) were assessed as demonstrating overall high methodological confidence and 11 studies (55%) as moderate confidence. No studies were assessed as low confidence. Studies of moderate confidence most commonly demonstrated limitations in confounding adjustment (Q6), participant recruitment transparency (Q3), and external generalisability (Q10). No studies were excluded on the basis of methodological confidence; all 20 were retained in the narrative synthesis, with appraisal findings considered during interpretation of findings.

Note: These ratings reflect structured qualitative appraisal using the CASP checklist as a qualitative tool. No numerical aggregate score was assigned to any study, in accordance with CASP Programme guidance. Overall confidence ratings reflect the pattern of domain-level assessments across Q1–Q10 and are not derived from any numerical threshold.
